# Enhancing Knowledge Retention in Medical Education Through Escape Box Games

**DOI:** 10.7759/cureus.82634

**Published:** 2025-04-20

**Authors:** Anya Ramsamooj, Jean Shanaa, Ethan Bernstein, Robert Augustynski, Nathaniel A Sands, Michayla Mabourakh, Hannah Chang, Jennifer Gullo

**Affiliations:** 1 Pediatrics, California Northstate University College of Medicine, Elk Grove, USA; 2 Orthopedic Surgery, California Northstate University College of Medicine, Elk Grove, USA; 3 Radiology, California Northstate University College of Medicine, Elk Grove, USA; 4 Emergency Medicine, California Northstate University College of Medicine, Elk Grove, USA; 5 Dermatology, California Northstate University College of Medicine, Elk Grove, USA

**Keywords:** escape box, gamification, medical education, pre-clinical learning, retention

## Abstract

Background: Gamification in medical education is a novel strategy to enhance student learning and engagement that is growing in popularity. One such game is an escape box, where players solve a series of challenges and riddles within a limited time to unlock a box or escape a virtual scenario. Building on the ongoing success of game-based learning, this study aims to expand on the use of games in medical school curricula by implementing an escape box-themed game as an effective strategy to teach pre-clinical science to first-year medical students.

Methods: The study, approved by the California Northstate University Institutional Review Board, involved first-year medical students divided into two groups: those who participated in the escape box game and lectures and those who attended lectures only. The study was conducted over three phases: a pre-game survey, the escape box game, and a post-game survey. Overall, the surveys included four questions that assessed confidence in renal medical concepts, three questions that assessed student engagement with the escape box game, and five questions that assessed personality traits based on the Big-Five OCEAN personality model.

Results: A total of 72 students participated, with 65 completing both pre- and post-game surveys. The final analysis included 40 students in the escape box, plus the lecture group, and 25 students in the lecture-only group. The escape box group of students found the escape box-themed game to be more stimulating and more interesting than traditional lecture, and for most students, it enhanced engagement beyond previous game-based learning methods. In addition, the overall increase in pre- versus post-game confidence was higher, but statistically insignificant in the escape box group. However, for one question regarding dialysis access, there was a statistically significant increase in confidence of 1.261 ± 0.13 for the escape box group and 0.763 ± 0.272 for the lecture-only group (p = 0.00434). No correlation was found between personality traits and an increase in confidence after escape box game learning.

Conclusion: Students found the escape box-themed game substantially more engaging than traditional lectures. There is a strong potential for its use to increase confidence in various other pre-clinical medical concepts. This study demonstrates that gamification of medical education topics, such as dialysis access, enhances student engagement, confidence, and retention (p = 0.00434). Future research should evaluate the longevity of knowledge retention and learning outcomes through longitudinal incorporation of game sessions and assessments throughout medical curriculum phases.

## Introduction

Consider a lecture hall filled with medical students, some following along diligently, but many others distracted by their computers, conversations, or other assignments. Maintaining student attention and engagement has become increasingly difficult, especially in the technological age. In response, some medical educators have begun incorporating new learning modalities to address these challenges. Gamification in medical education is a relatively new didactic strategy that has gained popularity. Gamification can be defined as the application of game mechanics in a nongaming context with the aim to enhance the process of learning and engagement with educational material [1]. The concept of gamification has historical roots in using game mechanics to facilitate learning and motivation, grounded in psychological and motivational theories such as Self-Determination Theory (SDT). SDT explains that gamified experiences engage learners by supporting their intrinsic psychological needs for autonomy, competence, and relatedness, thereby enhancing their motivation to learn [2].

Various educational fields have adopted gamification to increase student involvement and motivation, with substantial evidence pointing to its effectiveness [3]. Gamification transforms the traditional educational experience into a dynamic and engaging activity. Recent studies show that game-based learning significantly boosts motivation and participation among students while promoting teaching outcomes at multiple levels of the Kirkpatrick model [4]. At the reaction level (level 1), gamified approaches improve learner satisfaction, motivation, and engagement; at the learning level (level 2), gamification also aids knowledge translation and retention. However, more advanced outcomes such as behavioral change (level 3) or improved results at an organizational level (level 4) require further investigation, as limited studies demonstrate these higher-level impacts [4,5]. By incorporating game elements such as points, challenges, and rewards, learners are more likely to stay focused and invested in their studies [5]. This method not only makes learning enjoyable but also enhances critical thinking and problem-solving skills by presenting information in an interactive format. Furthermore, gamification caters to diverse learning styles, making education more inclusive and effective [6]. Ultimately, gamification has the potential to foster a deeper understanding and retention of material, improving overall educational outcomes while preparing students for real-world applications [5].

Recently, these principles have extended into medical education, where innovative strategies are being explored. Gamification strategies include the use of online question banks, which have shown improved undergraduate medical student engagement with microbiology topics and improved exam scores [7]. Kahoot, a live online question-answer game, is widely employed in first-year medical student curricula and has been shown to enhance focus, motivation, and retention of knowledge in immunology [8]. Jeopardy!-style review games have also shown improved engagement and knowledge retention among medical students [9]. Traditional board games, although less common, have been shown to improve undergraduate medical student performance and interest in clinical anatomy [10].

Building on the success of game-based learning, our study aims to test the hypothesis that escape box-themed games serve as an effective strategy for teaching pre-clinical science to first-year medical students. In this escape box model, participants work together following clues to solve a series of puzzles, riddles, and challenges within a set timeframe. When successfully deciphered, each exercise reveals a key or code combination that opens one of multiple locks securing a box. The goal of the students is to work together to be the first group to open all the locks and gain access to the box. Research by Cantwell et al. found that a virtual escape box design for emergency medicine clerkships increased engagement compared to traditional methods [11]. Thus, this approach has the potential to enhance student engagement, foster critical thinking, and improve retention of complex scientific concepts in medical education through experiential learning.

Through conducting our escape box-themed game, we hypothesize that group-based game learning is not only more engaging but may significantly enhance retention in medical education by transforming learning into an immersive and engaging experience. By incorporating medical scenarios, puzzles, and challenges relevant to the curriculum, students are motivated to actively participate and apply their theoretical knowledge in practical contexts, an experience often limited in traditional didactic learning. Similarly, the interactive nature of group-based escape games encourages critical thinking, problem-solving, and teamwork, mirroring real-world medical scenarios where quick decision-making is crucial [12]. We believe that this hands-on experience will not only reinforce key concepts but also boost long-term memory retention as students are more likely to remember information learned through active participation and emotional engagement with the material.

Ultimately, this study aims to evaluate the effectiveness of interactive "escape box" games in teaching pre-clinical science to first-year medical students. With many medical and nonmedical curricula shifting away from traditional textbook and lecture-based methods, we seek to demonstrate that interactive, group-based educational games like escape boxes are not only more engaging but also significantly enhance students' knowledge acquisition. A secondary objective was to investigate whether these innovative games are particularly beneficial for students with certain personality traits, such as extroversion or agreeableness, or if they are advantageous for all students regardless of personality type. Specifically, our study seeks to answer the following questions: Does an escape box provide a more engaging learning experience compared to traditional lectures or other educational games? Does incorporating an escape box alongside traditional lectures enhance student knowledge acquisition? Lastly, are collaborative educational games, such as escape box challenges, beneficial for all students, or do they primarily favor certain personality types?

## Materials and methods

Data collection

**Figure 1:**
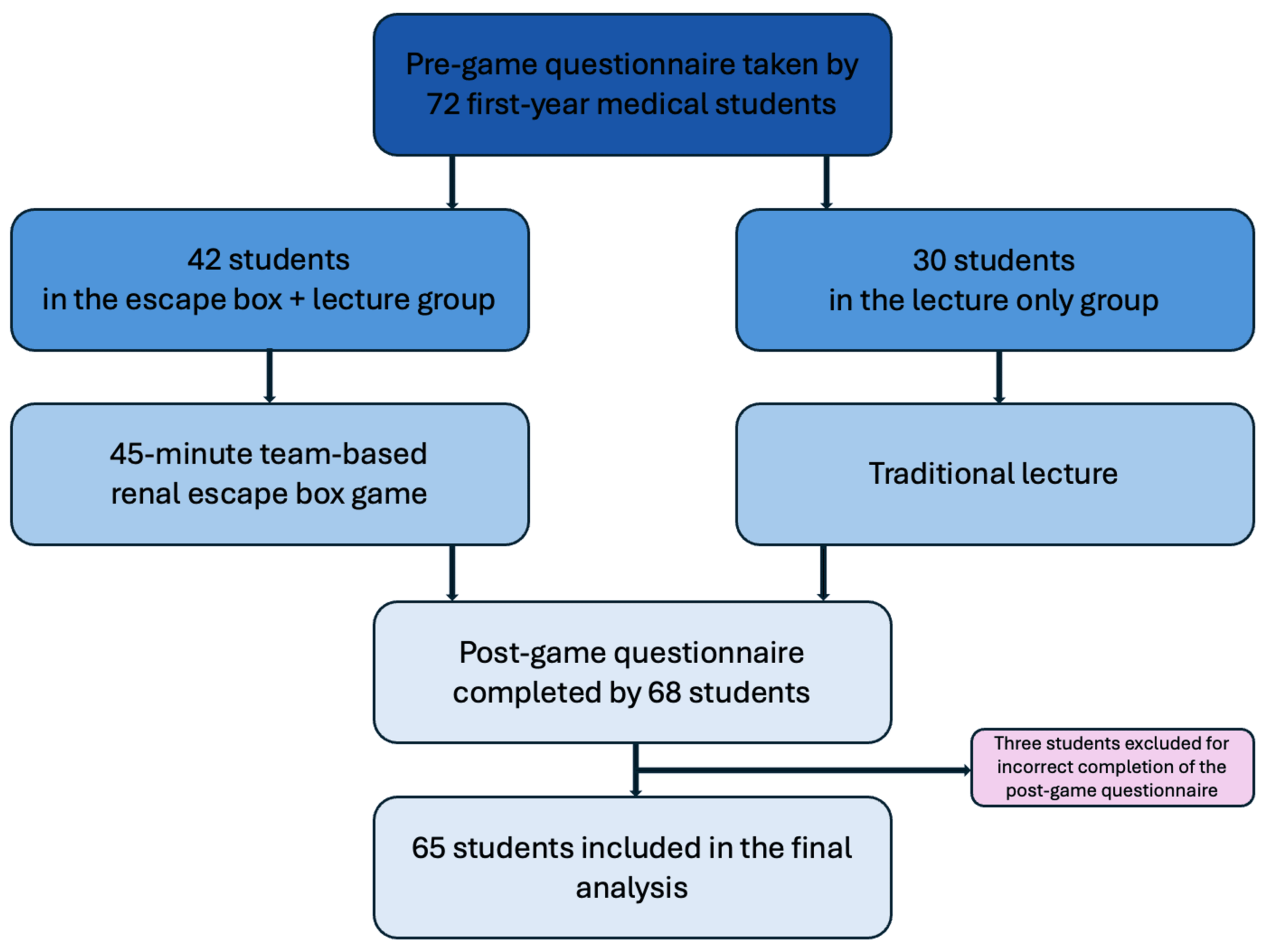
Study design algorithm

Following approval by California Northstate University’s (CNU) Institutional Review Board (protocol number = 2403-02-144), the study was conducted in three distinct phases: a pre-game questionnaire, the escape box game, and finally the post-game questionnaire (Figure 1). The questionnaire was pilot-tested by five students not contributing to the study to ensure clarity, reliability, and relevance before it was distributed to the larger sample. Inclusion criteria included first-year medical students from the California Northstate University College of Medicine (CNUCOM) Class of 2027 who were participating in the renal course block. First-year students were selected because they had no prior exposure to renal coursework, providing a consistent baseline, and they were the most readily accessible cohort. Exclusion criteria were students who incorrectly filled out the post-game questionnaire, specifically those who participated in the lecture-only group but answered questions designated for students who participated in both the lecture and escape box game groups. All other students were included in the final analysis. A convenience sample was selected based on those who decided to participate in the study and accurate completion of pre- and post-game questionnaires. This experimental study ran from March 18, 2024, to April 18, 2024, and compared students who engaged in both the escape box game and lectures to students who only engaged in lectures. The study followed guidelines provided by Warsinsky et al. in “Conceptual Ambiguity Surrounding Gamification and Serious Games in Health Care: Literature Review and Development of Game-Based Intervention Reporting Guidelines (GAMING)” [13]. The checklist can be found in the supplementary information.

The voluntary pre-game survey was administered online via SurveyMonkey at the beginning of the first-year medical students’ five-week-long renal block. The initial page featured a mandatory consent form, which students had to agree to in order to participate in the study. It included four questions regarding student confidence with foundational renal science and clinical renal-based medical concepts to measure baseline levels of knowledge of renal medicine. These knowledge questions were developed de novo by California Northstate College of Medicine Emergency Medicine and Nephrology faculty. Answer options included not confident, little confidence, confident, sufficiently confident, and highly confident. The pre-game survey also included five personality quantifying questions inspired by each of the Big Five OCEAN model of personality traits. Answer options included completely disagree, somewhat disagree, neutral, somewhat agree, and completely agree. 

**Figure 2:**
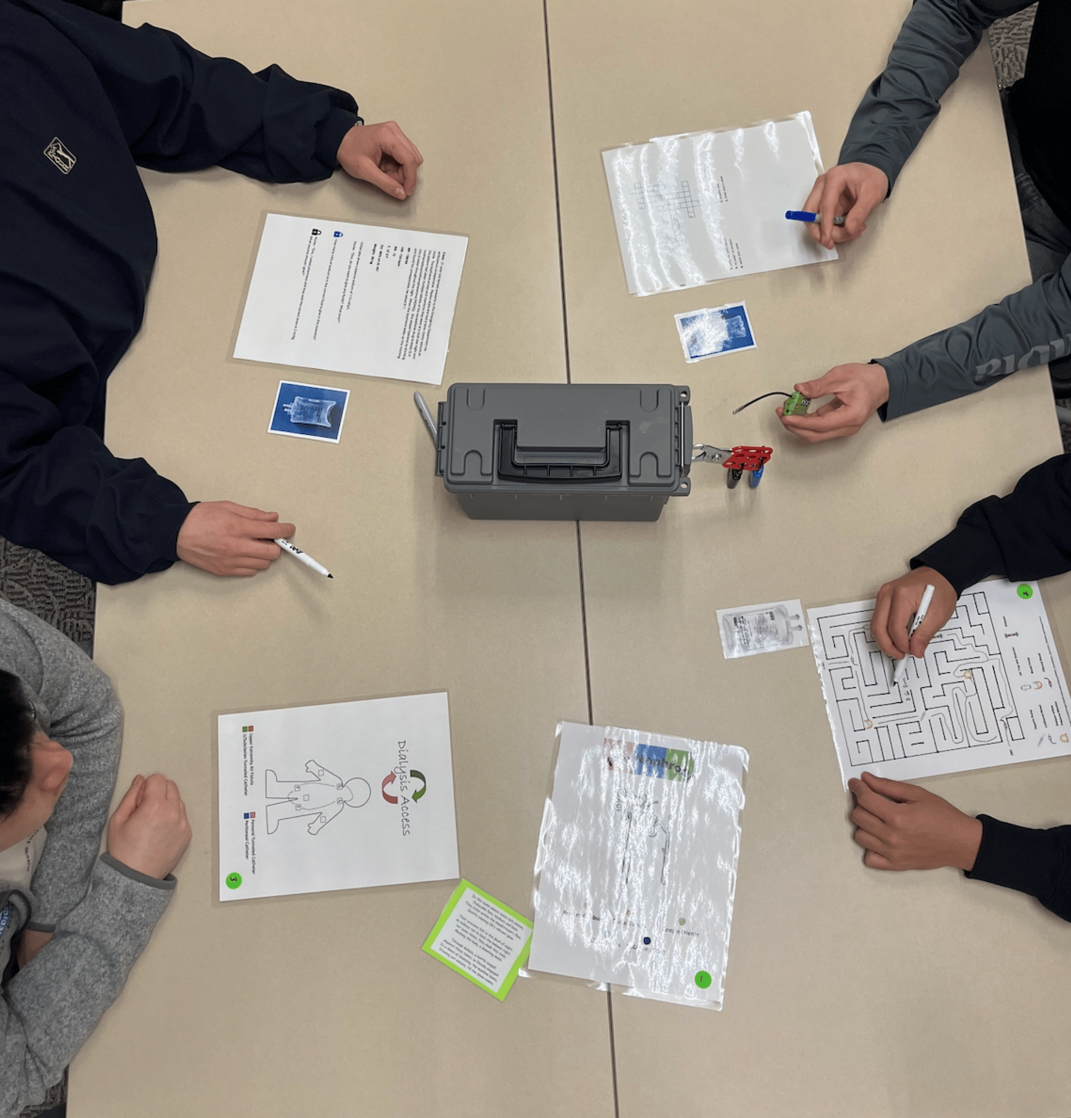
Students participating in the escape box game session

The escape box game, held in the first-year medical student lecture hall on the CNU campus, was an immersive, 45-minute team-based experience during the third week of the renal block. Designed to reinforce renal medicine concepts, the game required students to apply their knowledge creatively under time pressure. Working in teams of five, students navigated a series of puzzles and riddles, each covering different aspects of renal physiology, pathology, or treatment. For instance, one puzzle required completing a crossword on electrolyte and acid-base disorders, where the third letter of each word corresponded to a digit, ultimately forming the combination for one of the locks. An example of students participating in the game session is shown in Figure 2.

The puzzles were structured such that each correct answer revealed the code needed to unlock one of the ten physical locks securing the final box. The types of locks varied, from standard combination locks to more complex letter-based locks, adding a tactile element to the problem-solving process. To further heighten the competitive atmosphere, each team had a countdown timer visible in the lecture hall, motivating them to keep pace with their peers.

Guided by a specific CNU faculty member, who acted as game master, students could ask for a hint. Faculty encouraged teamwork and critical thinking, often using follow-up questions to guide students toward discovering the solution themselves. Teams raced to complete each lock's puzzle as quickly as possible, aiming to be the first to unlock all ten locks and access the final box.

**Figure 3:**
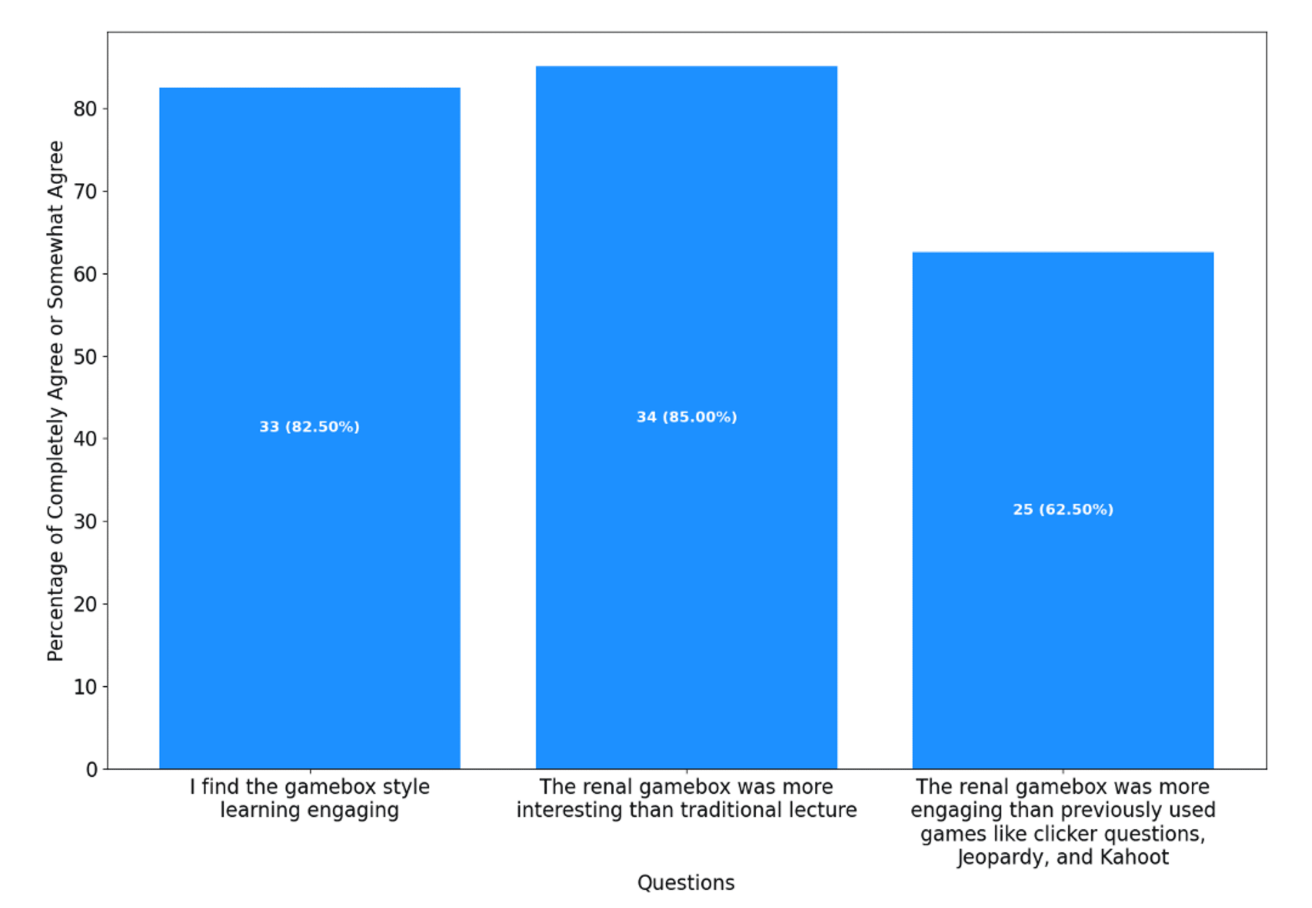
Number and percentage of students from the escape box game + lecture group who answered “completely agree” or “somewhat agree” to the engagement questions in the post-game survey (N = 40)

One week following the escape box game session, all students who completed the pre-game survey (both combined escape box game + lecture group and only lecture group) were invited to voluntarily complete the online post-lecture survey via SurveyMonkey. The post-game survey included the same previous four confidence questions as well as three questions which intended to quantify student engagement with the escape box game (Figure 3). The engagement questions were adapted from those used by Cantwell et al., and as a result, additional survey validity was deemed unnecessary. Answer options again included completely disagree, somewhat disagree, neutral, somewhat agree, and completely agree. Answers to all pre-game and post-game questions were graded on a Likert Scale from 1 (not confident or completely disagree) to 5 (highly confident or completely agree).

Statistical analysis

The average scores and standard error of the mean for each question were calculated for each group of students (escape box game and lecture versus lecture only) for both the pre-game and post-game surveys. Pre-game versus post-game confidence scores within each group were compared using a paired t-test. Additionally, the average change and standard error for confidence question scores were obtained and compared between the two groups using an independent sample t-test for each individual confidence question as well as for the average change in confidence as a whole. Lastly, the correlation between the average change in confidence and each personality score is reported as Kendall’s tau correlation coefficient and p-value. Type I error is set to 0.05.

## Results

Student impressions about escape box learning vs traditional lecture format

A total of 72 students participated in the study. Among them, 42 students participated in both the escape box game and in-person lecture, while 30 students attended the lecture only. Two students from the escape box group did not complete the survey. Additionally, survey results from three students in the lecture-only group were excluded because they incorrectly answered questions meant for the escape box participants in the post-game survey, in validating their data. Furthermore, two more students did not complete the post-game survey. The final number of participants included in the analysis was 65 students. 

Students who answered “completely agree” or “somewhat agree” were counted toward totals for engagement questions. A total of 84.62% of students found the gamebox style learning to be more engaging than the traditional lecture format. Moreover, 87.18% found the gamebox learning more interesting than the traditional lecture, while 64.1% of students found the gamebox learning more engaging than clicker questions, Jeopardy!, and Kahoot style learning, which had been employed in previous educational strategies (Figure 2).

Personality traits and confidence levels

There was no correlation found between personality traits and an increase in confidence after gamebox learning for any of the big five personality traits (Table 1).

**Table 1:** Correlation between personality traits and average change in confidence from pre-game survey to post-game survey for participants from both the escape box game + lecture group and lecture only group

		I consider myself extroverted	I consider myself agreeable	I consider myself neurotic	I consider myself conscientious	I tend to be open to new experiences
Average increase in confidence from pre-game survey to post-game survey	Kendall’s tau-b correlation coefficient	-0.0762	-0.0752	0.010	-0.110	-0.006
	p-value	p = 0.434	p = 0.461	p = 0.919	p = 0.286	p = 0.955
	N	65	65	65	65	65

Comparison of confidence between escape box learning and lecture-based learning

**Figure 4:**
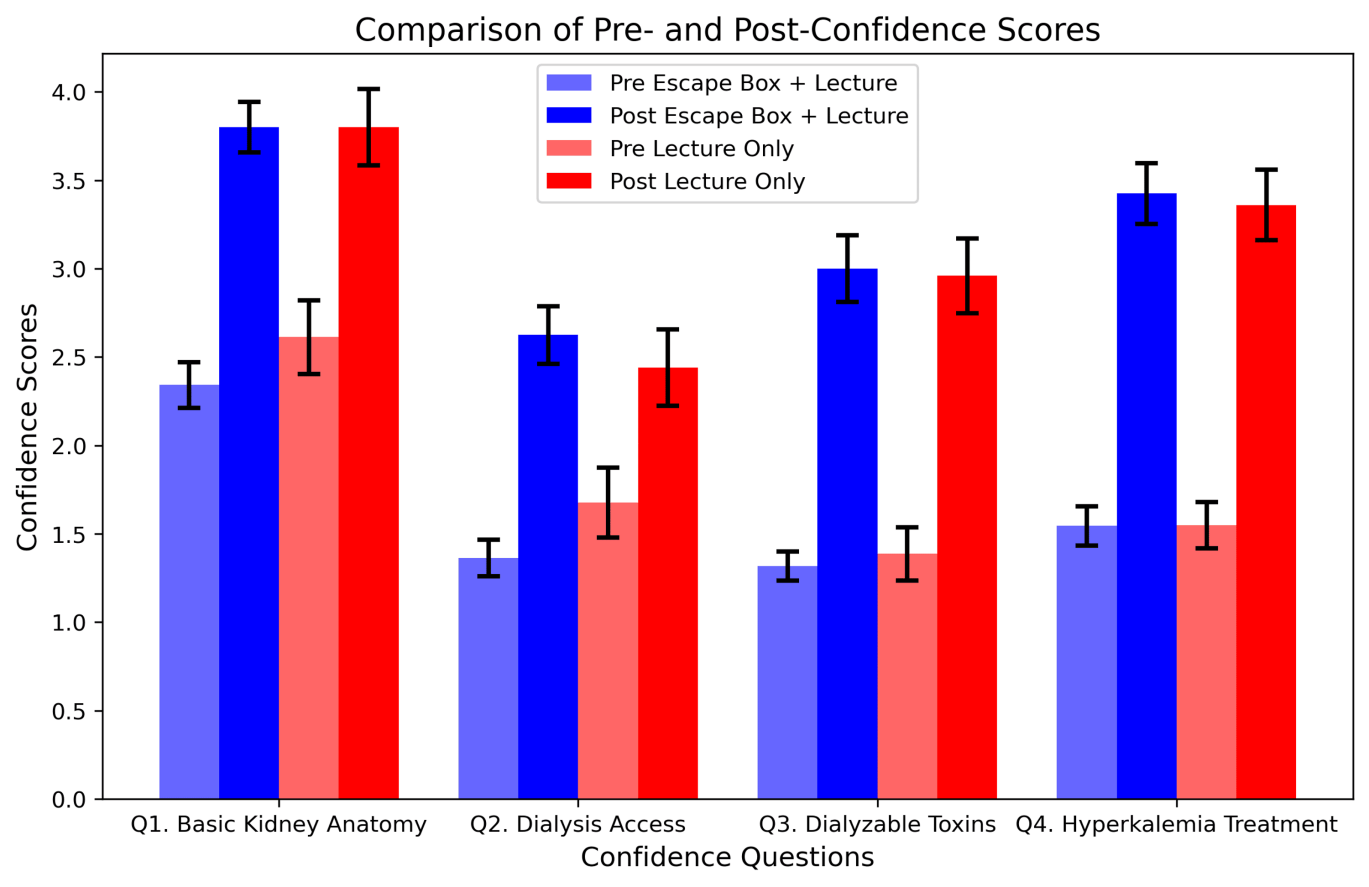
Side-by-side comparison of each group's pre- and post-confidence scores for each question

When looking at the escape box game with lecture-based learning and lecture-based learning only, respectively, there was a significant increase in the average total confidence score at the beginning versus at the end of the block for both groups (p = 0.0008, p = 0.0051) (Table 2). The escape box and lecture group began the block with an average confidence score of 1.7 ± 0.1 and ended the block with an average confidence score of 3.2 ± 0.17. The lecture-only group began the block with an average confidence score of 1.806 ± 0.17 and increased it to 3.14 ± 0.211 by the end (Figure 4). The surveys were conducted three weeks apart.

**Table 2:** Per question and average pre-game vs post-game scores for escape box + lecture and lecture-only groups

	N	Timing	Q1: How would you rate your confidence with basic kidney anatomy?	Q2: How would you rate your confidence on the various types of dialysis access?	Q3: How would you rate your confidence with your knowledge of dialyzable toxins?	Q4: How would you rate your confidence with the treatment of hyperkalemia?	AVG	t-test
Escape box game + lecture	40	Pre-game confidence score	2.342 ± 0.13	1.364 ± 0.104	1.318 ± 0.082	1.545 ± 0.112	1.664 ± 0.107	p = 0.0008
		Post-game confidence score	3.8 ± 0.144	2.625 ± 0.163	3 ± 0.189	3.425 ± 0.171	3.213 ± 0.167	
Lecture only	25	Pre-game confidence score	2.613 ± 0.208	1.677 ± 0.198	1.387 ± 0.15	1.548 ± 0.131	1.806 ± 0.172	p = 0.0051
		Post-game confidence score	3.8 ± 0.216	2.44 ± 0.216	2.96 ± 0.212	3.36 ± 0.199	3.14 ± 0.211	

Overall, there was a higher increase in confidence for the escape box + lecture group for all four questions. However, only question two saw a significant increase in confidence, which saw a change in confidence of 1.261 ± 0.13 for the combined group and 0.763 ± 0.272 for the lecture-only group (p = 0.00434). Similarly, the average total increase in confidence for the escape box + lecture group was higher than the lecture-only group, but not significant (Table 3).

**Table 3:** Per question and average change in confidence for escape box + lecture and lecture-only groups

	Escape box game + lecture (N = 40)	Lecture only (N = 25)	t-test
Q1: How would you rate your confidence with basic kidney anatomy?	1.368 ± 0.18	1.187 ± 0.19	p = 0.235
Q2: How would you rate your confidence on the various types of dialysis access?	1.261 ± 0.133	0.763 ± 0.272	p = 0.00434
Q3: How would you rate your confidence with your knowledge of dialyzable toxins?	1.682 ± 0.172	1.573 ± 0.199	p = 0.412
Q4: How would you rate your confidence with the treatment of hyperkalemia?	1.88 ± 0.16	1.812 ± 0.189	p = 0.476
Average change in confidence	1.548 ± 0.16	1.334 ± 0.2125	p = 0.230

## Discussion

When used to teach first-year medical students basic renal concepts, an escape box-themed game proved to be engaging and has the potential to enhance learning. Nearly all of the students who participated in the renal escape box game found it to be an engaging form of learning, more so than the engagement level of traditional lecture-style learning. Additionally, most of the students in the escape box group found that this form of game-based learning was more engaging than previously used game-based learning strategies, such as in-class clicker questions, Kahoot, and Jeopardy!-like games. This may be attributed to the fact that, unlike Kahoot and other Jeopardy!-style games, which are primarily individual in nature and emphasize rapid responses to multiple-choice questions, the escape box game fosters collaborative problem-solving around more complex and cognitively demanding tasks, which may enhance long-term retention.

The use of an escape box-themed game is relatively new in terms of the gamification of medical education. Previous work has found that escape room-themed games are most often used to evaluate or foster knowledge, rather than introduce new ideas [14]. One study examined the use of escape room stations to teach pre-clinical medical students about the basics of vascular surgery and found that participating students both enjoyed the game and thought that it helped solidify what they had learned during the study [15]. Only one of the students had prior knowledge of what was being taught in this study, thus allowing these escape room stations to be used to teach concepts rather than reinforce them. Another study performed on undergraduate students in a laboratory setting found that most students thought that the escape room lab was effective at reviewing previously learned laboratory techniques [16]. The escape box game employed in this study had distinct puzzles that taught new concepts, as well as others that reinforced concepts that were previously taught in lecture. 

Across all four confidence questions, students who participated in the renal game-box group were found to have greater increases in confidence compared to students in the lecture-only group. While the average change in confidence between the escape box game + lecture group and the lecture-only group was not significantly different (p = 0.230), this trend suggests that the escape box may be helpful as a supplement to traditional lecture. We believe the lack of significance is largely due to the substantial variability in individual responses to learning material. For example, struggling with the escape box puzzles may have caused some students to feel stressed about their grasp of the material, while others might have experienced confidence boosts simply from engaging with the content. Of note, the escape box + lecture group did experience a significant increase in confidence (p = 0.00434) for question two regarding knowledge of dialysis access. This difference may be due to the fact that there was an individual puzzle in the escape box that specifically covered dialysis access. In contrast to the other topics measured, dialysis access was not explicitly covered in the traditional lecture content of the renal course. This implies that an escape box game may also be effective in teaching new concepts to students. Future iterations of this escape box can be modified to further elucidate whether escape box learning is best suited for teaching or reinforcing concepts.

It has been previously suggested that gamification of a learning website has more of an impact on individuals with more extraverted personalities [17]. Additionally, it has been suggested that individuals with less neurotic personality traits may not benefit from gamification elements [18]. While our study did not show any correlation between personality traits and confidence, it is still important to note that game-based learning may not be beneficial to all students in a cohort. Future studies specifically regarding the application of an escape box modality should expand on its use across different personalities and how it should be best applied to the medical school curriculum.

Limitations

One limitation of this study lies in the fact that not all participating students took the pre- and post-intervention surveys at the same time point, as the surveys were available for completion over several days. Future studies could enhance consistency by requiring all students to complete the surveys simultaneously or by limiting the survey availability window to just one day. Moreover, this study measured the efficacy of the escape box using students’ confidence with various renal-based medical concepts. A more objective measure of knowledge retention could involve administering a pre- and post-game knowledge assessment with specific renal questions that have only one correct answer, leading to a more accurate assessment of this variable. Lastly, increasing the sample size to include the entire cohort could result in a larger and potentially statistically significant change in confidence for the game + lecture group. Due to the limited sample sizes in our groups (40 and 25 participants, respectively), we were unable to validate our hypothesis that participating in the escape box would significantly improve students' knowledge acquisition. Data saturation was not reached in this study due to the relatively low number of subjects. 

Future directions

Previous work has found that the use of an escape box was engaging for third- and fourth-year medical students and was determined to be an effective method for learning as measured on a Likert scale [10]. As our study mainly focused on pre-clinical concepts and basic science, it builds on this previous work and suggests that escape box games can be applied to both the pre-clinical and clinical phases of medical education. As such, future studies on escape box-themed games could be conducted with both pre-clinical and clinical concepts to solidify where gamification of this kind is most useful. This study only focused on the “reaction” level of the Kirkpatrick model, as participant engagement and subjective confidence levels were elevated. Thus, future studies can expand on this and assess the learning and behavior levels of the Kirkpatrick model; the results level can be further evaluated if an escape box-themed game is fully implemented into the medical curricula [19]. Rather than using subjective measures like confidence to evaluate efficacy, future iterations of an escape box game can use more objective measures, such as correlation with course exam scores or pre- and post-game quizzes. Future escape box games can also be employed at various time points throughout a course to determine when it can be most effective and allow for a pre-assessment before the course starts and a post-assessment after the course has concluded. Lastly, future studies could address this study’s limitations by expanding the sample size, increasing the number of game sessions, or evaluating long-term knowledge retention more comprehensively.

## Conclusions

The implementation of an escape box-themed game for teaching first-year medical students basic renal concepts demonstrated significant engagement and potential for enhancing learning. Respondents found the game more interesting than traditional lecture methods and more engaging than other game-based strategies, such as audience response questions, Kahoot, and Jeopardy!-like games. These results suggest that escape box learning may facilitate the assimilation of new content and reinforce learned concepts of the renal system and in medical education overall. Moreover, we believe that greater emphasis should be placed on these types of games, as the skills developed through collaborative activities like the escape box are highly transferable to real-world medical practice, particularly in emergency settings where rapid, team-based problem-solving is critical. Although the study did not find a significant difference in overall confidence between the escape box and lecture-only groups, it did show a notable increase in confidence regarding specific topics like dialysis access, which were not covered in traditional lectures. No correlation was found between specific personality traits and increased confidence in knowledge, indicating that no specific personality type is needed to benefit from educational games. Our findings indicate that the incorporation of escape box learning can add active learning and team-building improvements to pre-clinical education. Future research should address the impact of escape box learning on medical student knowledge retention over extended periods and across other medical topics and compare game-based strategies to other teaching methodologies such as case-based learning.

## Appendices

Questionnaires

Pre-game survey part 1: questions on knowledge regarding basic renal concepts

How would you rate your confidence with basic kidney anatomy?

●      1 = not confident

●      2 = little confidence

●      3 = some confidence

●      4 = confident

●      5 = highly confident

How would you rate your confidence with dialysis access?

●      1 = not confident

●      2 = little confidence

●      3 = some confidence

●      4 = confident

●      5 = highly confident

How would you rate your confidence with dialyzable toxins?

●      1 = not confident

●      2 = little confidence

●      3 = some confidence

●      4 = confident

●      5 = highly confident

How would you rate your confidence with the treatment of hyperkalemia?

●      1 = not confident

●      2 = little confidence

●      3 = some confidence

●      4 = confident

●      5 = highly confident

Pre-game survey part 2: assessment of personality types

How much do you agree with the following statement: I consider myself extraverted.

●      1 = completely disagree

●      2 = somewhat disagree

●      3 = neutral

●      4 = somewhat agree

●      5 = completely agree

How much do you agree with the following statement: I consider myself agreeable.

●      1 = completely disagree

●      2 = somewhat disagree

●      3 = neutral

●      4 = somewhat agree

●      5 = completely agree

How much do you agree with the following statement: I consider myself neurotic (emotionally unstable)

●      1 = completely disagree

●      2 = somewhat disagree

●      3 = neutral

●      4 = somewhat agree

●      5 = completely agree

How much do you agree with the following statement: I consider myself conscientious.

●      1 = completely disagree

●      2 = somewhat disagree

●      3 = neutral

●      4 = somewhat agree

●      5 = completely agree

How much do you agree with the following statement: I tend to be open to new experiences.

●      1 = completely disagree

●      2 = somewhat disagree

●      3 = neutral

●      4 = somewhat agree

●      5 = completely agree

Would you consider yourself an introvert or extrovert?

Introvert

Extrovert

Post-game survey: questions on lecture vs lecture + renal gamebox:

Did you attend the renal gamebox session?

Yes

No

Did you watch renal weeks 1 and 2 lectures (in-person or via Mediasite)?

Yes

No

How engaging do you find traditional lecture-style learning?

●      1 = not at all engaging

●      2 = somewhat engaging

●      3 = very engaging

How much do you agree with the following statement: I understand the content from renal weeks 1 and 2?

●      1 = completely disagree

●      2 = somewhat disagree

●      3 = neutral

●      4 = somewhat agree

●      5 = completely agree

How would you rate your confidence with basic kidney anatomy?

●      1 = not confident

●      2 = little confidence

●      3 = some confidence

●      4 = confident

●      5 = highly confident

How would you rate your confidence with dialysis access?

●      1 = not confident

●      2 = little confidence

●      3 = some confidence

●      4 = confident

●      5 = highly confident

How would you rate your confidence with dialyzable toxins?

●      1 = not confident

●      2 = little confidence

●      3 = some confidence

●      4 = confident

●      5 = highly confident

How would you rate your confidence with the treatment of hyperkalemia?

●      1 = not confident

●      2 = little confidence

●      3 = some confidence

●      4 = confident

●      5 = highly confident

Additional questions for renal game participants only:

How engaging did you find the gamebox style learning?

●      1 = not at all engaging

●      2 = somewhat engaging

●      3 = very engaging

 How much do you agree with the following statement: The renal gamebox was more interesting than traditional lecture.

●      1 = completely disagree

●      2 = somewhat disagree

●      3 = neutral

●      4 = somewhat agree

●      5 = completely agree

How much do you agree with the following statement: The renal gamebox was more engaging than previously used games like clicker questions, Jeopardy!, and Kahoot.

●      1 = completely disagree

●      2 = somewhat disagree

●      3 = neutral

●      4 = somewhat agree

●      5 = completely agree

**Table 4:** Game-based intervention reporting guidelines-short version

#	Topic	Description	Exemplary/explanatory statements
1	Conceptual focus	(a) Decide which concepts (i.e., gamification, serious games, etc.) best reflect the interventions you want to investigate	Gamification
		(b) Clearly state early in the paper which core concepts (i.e., gamification, serious games, etc.) you focus on in your study, and why	“Building on the success of game-based learning, our study aims to test the hypothesis that escape box-themed games serve as an effective strategy for teaching pre-clinical science to first-year medical students. In this model, participants work together following clues to solve a series of puzzles, riddles, and challenges within a set timeframe to decipher lock combinations fitted to a box”
		(c) Supply only metadata (e.g., title, keywords) that corresponds to your core concepts	Medical education, escape box, gamification, pre-clinical learning, retention
2	Contribution	(a) Decide which research stream within the focused concepts your study contributes to	Our study focuses on the use of gamification in pre-clinical medical education and builds specifically off of previous studies (Cantwell et al., 2022) using escape box-themed games to reinforce medical concepts
		(b) Report which research streams your study contributes to and which criteria the decision for research streams was based on	We contribute to the use of escape box-themed games in first-year medical students, as compared to previous work where escape box-like games were used in clinical/clerkship years.
		(c) Clarify your study's contributions to the chosen research streams	“The implementation of an escape box-themed game for teaching first-year medical students basic renal concepts demonstrated significant engagement and potential for enhancing learning. The game proved to be more engaging than traditional lecture methods and other game-based strategies, such as audience response questions, Kahoot, and Jeopardy!-like games”
		(d) Clarify your study's contributions to solving a problem or need in practice or society	“These results suggest that escape box learning may facilitate the assimilation of new content and reinforce learned concepts of the renal system and in medical education overall.”
		(e) Report to which extent observed positive and negative outcomes can be attributed to your game-based approach. If possible, narrow down the attribution of outcomes based on individual components of your game-based approach (e.g., game elements)	“When used to teach first-year medical students basic renal concepts, an escape box-themed game proved to be both engaging and has the potential to enhance learning. Nearly all of the students who participated in the renal escape box game found it to be an engaging form of learning, more so than the engagement level of traditional lecture-style learning. Additionally, most of the students in the escape box group found that this form of game-based learning was more engaging than previously used game-based learning strategies, such as in-class clicker questions, Kahoot, and Jeopardy!-like games”
3	Mindfulness about related concepts		
3.1	Introduction and use of related concepts	(a) Make efforts to identify possibly related concepts prominent in the context of your study	Prominent related concepts for gamification in medical education include personality type and learning style; however, our study did not demonstrate that there was a correlation between personality traits and changes in confidence with renal concepts
		(b) Mention only those related concepts that are substantive for your study	Prior to beginning the renal escape box game, participants were given an overview of the objective of the escape box and the goal to solve puzzles that unlock a series of locks to ultimately unlock the final box
		(c) Be mindful about nuanced terms in the domain of any introduced concept and use established vocabulary precisely	n/a
		(d) Avoid using related concepts interchangeably. If you use an umbrella term, specify which terms it comprises, and clarify why you introduce it	n/a
3.2	Insights from extant literature	(a) Be mindful about conceptual ambiguities when drawing on literature about game-based interventions	n/a
		(b) Do not presume easy transferability of insights from one concept to another	n/a
		(c) Specify precisely what you draw from literature and why these insights are applicable to your study	“Gamification in medical education is a relatively new didactic strategy that has gained popularity. Gamification can be defined as the application of game mechanics in a non-gaming context with the aim of enhancing the process of learning and engagement with educational material (Busch 2014). The concept of gamification has historical roots in using game mechanics to facilitate learning and motivation, grounded in psychological and motivational theories such as Self-Determination Theory (SDT). SDT explains that gamified experiences engage learners by supporting their intrinsic psychological needs for autonomy, competence, and relatedness, thereby enhancing their motivation to learn (Deterding et al., 2011). Various educational fields have adopted gamification to increase student involvement and motivation, with substantial evidence pointing to its effectiveness (Alk et al., 2013). Gamification transforms the traditional educational experience into a dynamic and engaging activity”
4	Individual concept definitions		
4.1	Definition inspiration	(a) Familiarize yourself with definitions for a concept provided by the extant literature	Escape education: a systematic review on escape rooms in education (Veldkamp et al., 2020) was reviewed to better understand the previous use of escape-themed games in education
		(b) Decide whether a concept definition from extant literature is applicable for your research, or if you need a self-developed definition	Gamification as defined in extant literature: “Gamification can be defined as the application of game mechanics in a non-gaming context with the aim to enhance the process of learning and engagement with educational material (Busch 2014)”
4.2	Definition of concepts	(a) Explicitly define each introduced concept independently in a principal clause.	Gamification: “Gamification can be defined as the application of game mechanics in a non-gaming context with the aim to enhance the process of learning and engagement with educational material (Busch 2014).” Escape box game: A game involving a serious of puzzles that must be solved to unlock a set of combination locks that will ultimately unlock a bigger locked box
		(b) Explicitly distinguish each introduced related concept pairwise to at least to your core concepts; better even to all related concepts	Gamification in this study differs from serious games in that the escape box game was used to reinforce renal concepts that were previously covered in lecture. Rather than teaching a new concept as a serious game would, the escape box game employed here was used to determine whether students confidence increased and whether they found this more engaging than previously employed gamification techniques
4.3	Definitions from extant literature	If definitions are taken from extant literature…	
		(a) Make efforts to identify the original source of a definition	“The concept of gamification has historical roots in using game mechanics to facilitate learning and motivation, grounded in psychological and motivational theories such as Self-Determination Theory (SDT). SDT explains that gamified experiences engage learners by supporting their intrinsic psychological needs for autonomy, competence, and relatedness, thereby enhancing their motivation to learn (Deterding et al., 2011)”
		(b) Include an explicit reference to the source of a definition directly following the definition.	“Building on the success of game-based learning, our study aims to test the hypothesis that escape box-themed games serve as an effective strategy for teaching pre-clinical science to first-year medical students. In this model, participants work together following clues to solve a series of puzzles, riddles, and challenges within a set timeframe to decipher lock combinations fitted to a box”
4.4	Self-developed definitions	If any definition for a concept is self-developed...	
		(a) Make sure to adhere to good definition design	n/a
		(b) Clarify from which views your self-developed definition emerged	Description of the gamification and escape box-themed game used in this study: “The escape box game, held in the first-year medical student lecture hall on the CNU campus, was an immersive, forty-five-minute team-based experience during the third week of the renal block. Designed to reinforce renal medicine concepts, the game required students to apply their knowledge creatively under time pressure. Each team of five students worked through a series of puzzles and riddles, each designed to cover different aspects of renal physiology, pathology, or treatment. The puzzles were structured such that each correct answer revealed the code needed to unlock one of the ten physical locks securing the final box. The types of locks varied, from standard combination locks to more complex letter-based locks, adding a tactile element to the problem-solving process. To further heighten the competitive atmosphere, each team had a countdown timer visible in the lecture hall, motivating them to keep pace with their peers. Guided by CNU faculty members, who acted as game masters, students could ask for a hint. Faculty encouraged teamwork and critical thinking, often using follow-up questions to guide students toward discovering the solution themselves. Teams raced to complete each lock's puzzle as quickly as possible, aiming to be the first to unlock all ten locks and access the final box”
4.5	Multiple definitions for a single concept	If multiple definitions for a single concept are provided …	n/a
		(a) State clearly, which definitions are applied in the study, and why	n/a
		(b) apply the chosen definition(s) consistently	n/a
